# Regulatory time-kill sequencing maps antibiotic-specific transcriptional control of *Escherichia coli* persistence at genome scale

**DOI:** 10.3389/fmicb.2026.1950209

**Published:** 2026-09-24

**Authors:** Alden Filko, Yichao Han, Fuzhong Zhang

**Affiliations:** 1 Department of Energy, Environmental and Chemical Engineering, Washington University in St. Louis, Saint Louis, MO, United States; 2 Energy and Environment Directorate, Pacific Northwest National Laboratory, Richland, WA, United States; 3 Division of Biological and Biomedical Sciences, Washington University in St. Louis, Saint Louis, MO, United States; 4 Institute of Materials Science and Engineering, Washington University in St. Louis, Saint Louis, MO, United States

**Keywords:** antibiotic persistence, antibiotic response, highthroughput analysis, systems biology, time kill assay and antibacterial activity

## Abstract

Bacterial persistence enables a phenotypically tolerant subpopulation to survive lethal antibiotic exposure, contributing to the recalcitrance of chronic infections and facilitating the evolution of heritable resistance. Despite its importance, a systems-level, quantitative understanding of how transcriptional regulation shapes persistence kinetics has remained limited, largely because conventional time-kill assays are inherently low-throughput. Here, we develop Regulatory Time-Kill Sequencing (RTK-seq), a high-throughput functional genomics strategy that combines a pooled CRISPR interference (CRISPRi) library targeting transcription factors with time-resolved antibiotic selection and deep barcode sequencing. RTK-seq simultaneously resolves multiphasic killing trajectories for 174 *Escherichia coli* transcriptional regulators over five orders of magnitude in survival. The resulting profiles recapture known persistence pathways and identify previously unrecognized regulators, including *rcsA*, *narP*, and *csgD*, that modulate distinct phases of antibiotic killing. Additionally, applying RTK-seq across multiple antibiotic classes reveals a highly condition-dependent regulatory landscape, demonstrating that persistence is governed not by a single universal program but by drug-specific metabolic and stress responses. Overall, RTK-seq offers a scalable, versatile strategy for quantitatively mapping microbial survival networks and identifies targets for anti-persister therapies.

## Introduction

Antibiotic treatment often yields a biphasic killing response: an initial rapid decline in viable cells followed by a markedly slower phase in which a small subpopulation survives for extended periods. This phenomenon, termed antibiotic persistence, reflects increased drug tolerance in a subset of cells within an otherwise genetically susceptible population (Balaban et al., 2019; Keren et al., 2004; Lewis, 2010). In contrast to heritable antibiotic resistance, which reduces susceptibility of the entire population at a given drug concentration, tolerance and persistence describe the ability of cells to survive longer under antibiotic exposure without changes in the minimum inhibitory concentration (MIC). Persistent cells are thought to underlie many recurrent and recalcitrant infections that appear antibiotic-susceptible in standard clinical tests (Ojha et al., 2008; Mulcahy et al., 2010; Fauvart et al., 2011; Morrison et al., 2020), and they can facilitate the eventual evolution of resistance during repeated treatments (Levin-Reisman et al., 2017; Windels et al., 2019; Liu et al., 2020). Persistence is therefore a major contributor to the global antibiotic resistance crisis.

Despite intensive studies, our understanding of the systems-level regulation of persistence remains limited. Specific physiological states, including starvation, stationary phase, nutrient switch, and stress responses, can increase the frequency of tolerant or persistent cells within bacterial population (Pacios et al., 2020; Orman and Brynildsen, 2015; Jõers and Tenson, 2016; Balaban et al., 2013; Dewachter et al., 2021; Zhang et al., 2024; Hartline et al., 2022). Furthermore, specific molecular pathways—such as toxin-antitoxin (TA) modules, reactive oxygen species management, ppGpp accumulation (stringent response), heterogeneity in gene expression and metabolism, efflux pump activity, and epigenetic modifications—have all been shown to influence persistence levels (Zhang et al., 2024; Zhang et al., 2026; El Meouche and Dunlop, 2018; Huang et al., 2020; Kaspy et al., 2013; Schmitz et al., 2017; Radzikowski et al., 2016; Evans and Zhang, 2020).

At the genetic level, perturbation of individual genes (e.g., *relE* and *mazF*) has also been shown to affect cellular dormancy and antibiotic tolerance. These systems can inhibit growth through mechanisms such as disrupting DNA replication or translation (Singh et al., 2021; Gerdes and Maisonneuve, 2012; Page and Peti, 2016). Additionally, high-throughput approaches have begun to uncover genes associated with altered persistence (Mohiuddin et al., 2022). For example, genome-wide mutation libraries have revealed loci whose disruption alters survival (Girgis et al., 2012). Collectively, these studies have generated valuable gene targets. However, previous studies have largely focused on individual perturbations rather than systematically interrogating the broader regulatory networks. A deeper understanding of persistence requires perturbing these interconnected, redundant stress-response networks at a systems level to reveal epistatic interactions and emergent behaviors.

A central technical gap limiting such systematic studies is the lack of high-throughput, quantitative methods for characterizing persistence. Conventional persistence assays involve growing individual cultures, exposing them to antibiotics, sampling over time, removing the drug, and plating for colony-forming unit (CFU) quantification. Scaling this to hundreds or thousands of genetic backgrounds is prohibitively labor- and time-intensive. While some high-throughput screens have identified persistence-associated genes, they often rely on static single-endpoint assays that fail to capture the multi-phasic kinetic trajectories distinguishing true persistence from general tolerance. Time-resolved killing curves (time-kill assays) are the gold standard for resolving these dynamics but have not been scaled systematically across diverse genomic backgrounds in previous genome-wide studies. A high-resolution, time-resolved approach is therefore essential to map the epistatic topology of these networks and generate the continuous parameters needed for predictive computational modeling. This bottleneck currently prevents a comprehensive, genome-scale view of how regulatory networks shape antibiotic tolerance and persistence.

Transcription factors (TFs) provide an attractive entry point for systems-level dissection of persistence. TFs occupy key nodes in regulatory networks and collectively control the expression of most genes in the cell (Han et al., 2023). Perturbing TFs can efficiently influence the expression level of many individual genes, thereby affecting bacterial persistence. Recent advances in CRISPR-based technologies have enabled pooled TF knockdown (TFKD) libraries, in which the expression of each TF is repressed by dCas9 and specific sgRNA. When coupled with high-throughput phenotypic analysis, such as promoter activity measurement followed by sequencing, these approaches allow the regulatory functions of all TF’s to be examined in a single experiment (Han et al., 2023). Here, we repurpose this powerful high-throughput platform to quantify antibiotic killing curves after repression of each TF. We developed a strategy called Regulatory Time-Kill Sequencing (RTK-seq), which allowed us to identify multiple TFs whose knockdown increases or decreases the fraction of persistence cells by at least one order of magnitude, enabling us to dissect the systems-level regulation of persistence.

## Results

### A multiplexed platform for pooled transcription factor perturbation and persistence profiling

To investigate regulatory networks that govern persistence, we first created a library of TFKD *E. coli* strains. We used a previously constructed plasmid library containing 183 gRNAs that target the start of the coding region of every known *E. coli* TF (Han et al., 2023; Wang et al., 2018). This plasmid library was then transformed into the *E. coli* strain Fr-E01, which contains a genomically integrated dCas9 for gene repression (Figure 1A), thereby generating the TFKD strain library. This approach significantly represses 95% of the target TFs (Han et al., 2023). The lack of repression for the remaining 5% was mostly caused by their low expression levels, which makes further repression difficult to detect. To quantify the time-killed curve of every TFKD strain, we developed a method that combines population-level time-kill assays with high-throughput sequencing. Briefly, the pooled strain library was first grown to steady state in M9 glucose medium to maintain a defined, reproducible media, and then exposed to ampicillin at 100 μg/mL, a concentration well above its MIC (May et al., 2009) (Figure 1, step 1 and 2). Cultures were sampled at multiple time points following antibiotic treatment (Figure 1, step 3). At each time point, cells were washed to remove antibiotics and split into two fractions. One fraction was used for standard persistence assay, in which serial dilutions were plated to determine CFUs and generate a population-level time-kill curve (Figure 1, steps 4 and5). The other fraction was plated onto large agar plates with a minimum of 20,000 colonies (Figure 1, step 6). Colonies from each plate were pooled for plasmid DNA extraction to recover the gRNAs mixture corresponding to library members that survived to that time point (Figure 1, step 7).

**Figure 1:**
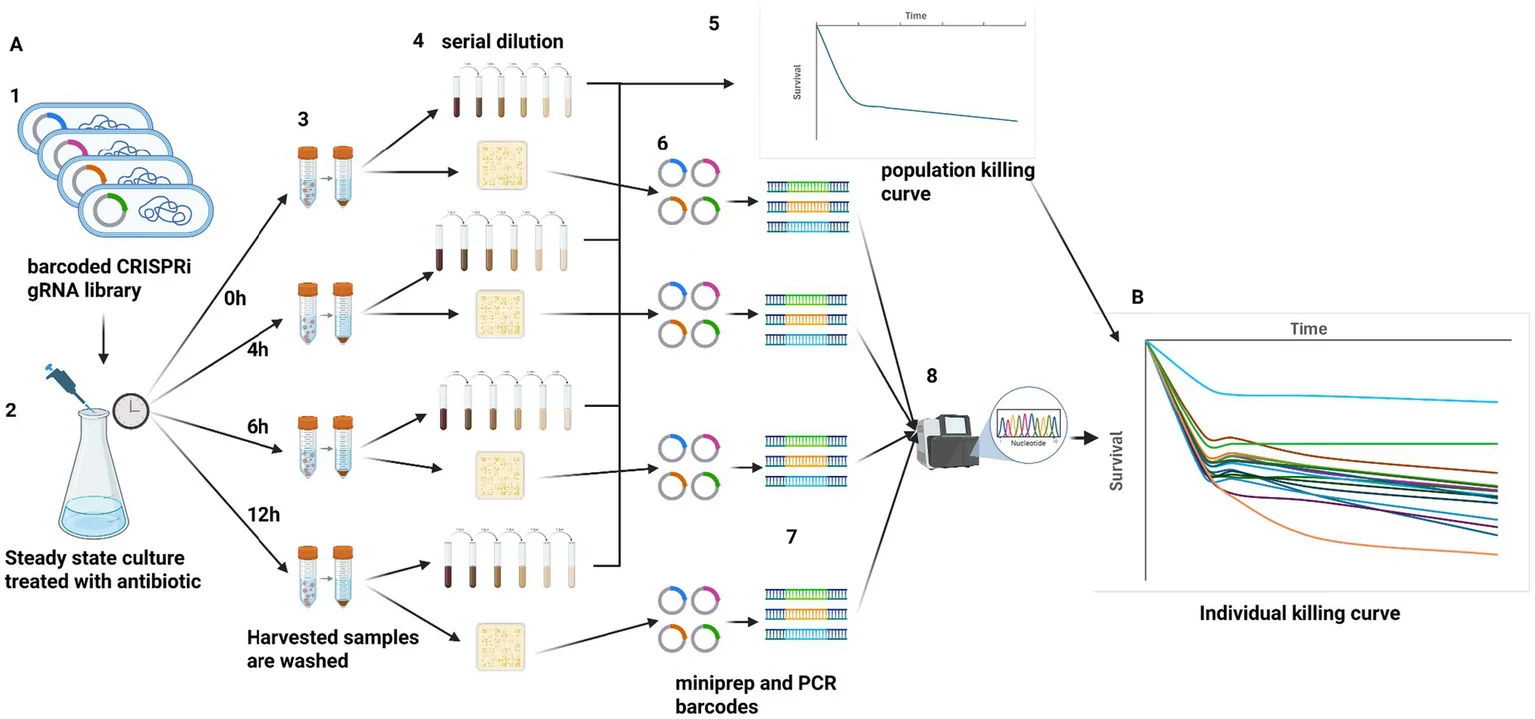
Workflow overview of RTK-seq. **(A)** Experimental workflow. (1) A pooled sgRNA library was transformed into *E. coli*, with each unique barcoded guide targeting a specific transcription factor. (2) Exponentially growing cultures were treated with an antibiotic. (3) At each time point, an aliquot was collected and washed with PBS buffer to remove the antibiotic. (4) Serial dilutions were made for colony-forming unit (CFU) counting. (5) CFU counts were used to generate the time-kill curve for the culture mixture. (6) At least 20,000 surviving cells from each time point were plated and grown into colonies, after which plasmids carrying the sgRNA barcodes were isolated. (7) Sample were indexed by PCR and pooled. (8) The DNA mixtures were quantified by sequencing. **(B)** Computational work. The population-level time-kill curve was combined with the relative abundance of each library member determined by sequencing to reconstruct an individual time-kill curve for every sgRNA in the library.

The gRNA mixtures collected at each time point were then uniquely barcoded by PCR and pooled for next-generation sequencing at a depth of 7.7 M reads per sample (Figure 1, step 8). Sequencing reads were used to determine the abundance of each gRNA at each time point. By integrating the relative abundance of each library member with the surviving fraction of the total population (from CFU measurements) (Equations 1–4), a time-resolved time-kill curve for each TF knockdown strain can be reconstructed (Figure 1B).

From a single pooled experiment, we generated survival curves for 174 TFKD strains under ampicillin treatment (Figure 2A, Supplementary Table 2). The method captured survival ratios over approximately five orders of magnitude. While 61% of the TFKD strains did not substantially alter persistence and clustered near the survival level of the negative control, which carried a non-targeting sgRNA (red line in Figure 2A), 5 TFKD (*hipB*, *mntR, rcsA, narP* and *oxyR*) strains showed more than a 10-fold higher increase in survival by 24 h of ampicillin treatment, and 12 TFKD strains (*sdiA, ihfB, csgD, torR, sgrR, caiF, acrR, exuR, flhC, ihfA, ompR, relB*) showed more than a 10-fold decrease in survival (Figure 2B).

**Figure 2:**
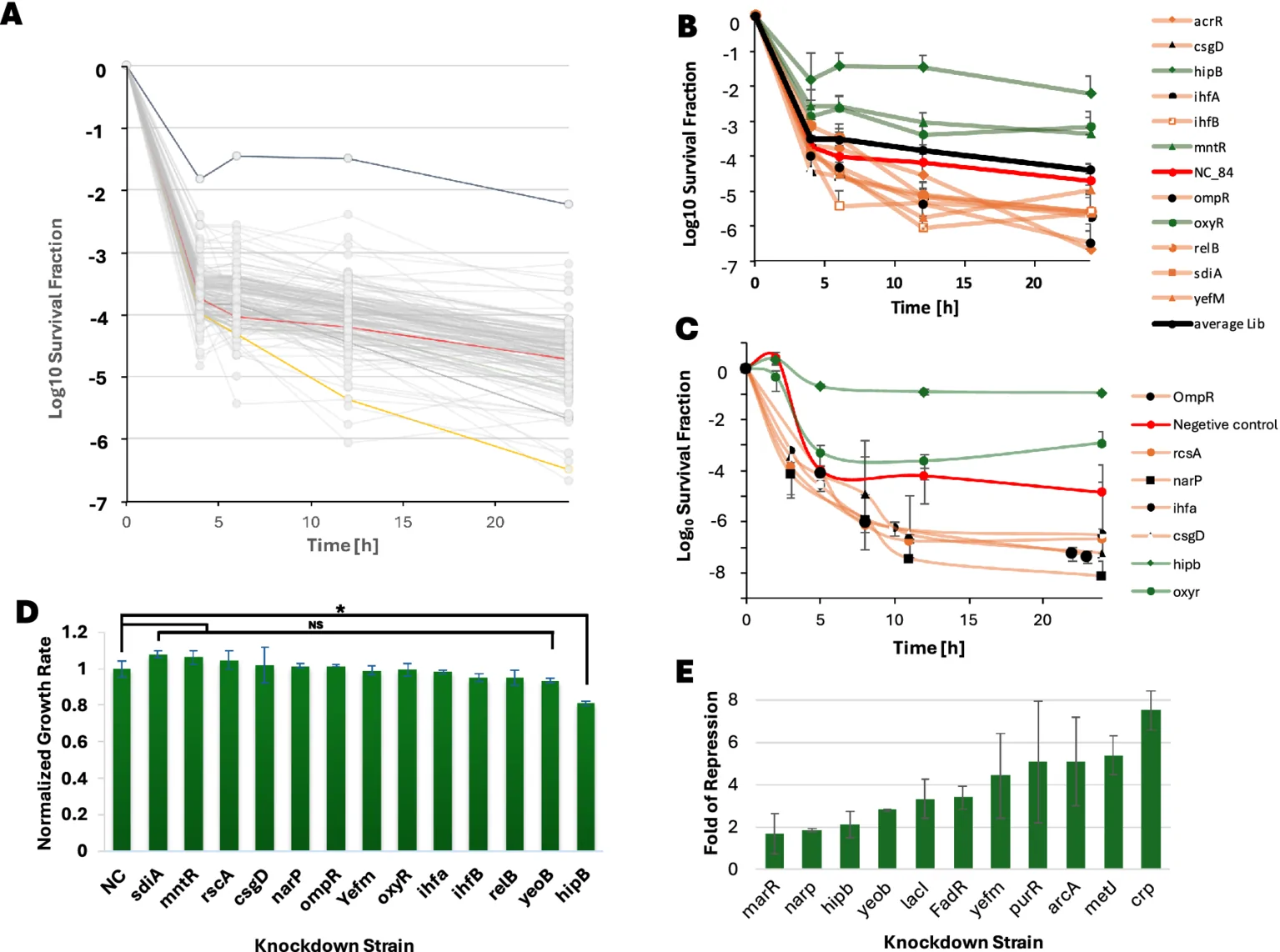
RTK-seq results and quality-control analyses. **(A)** Survival curves for all members of the RTK-seq library representing the average of biological triplicates. The negative control strain is highlighted in red, while *hipB* and *ompR* knockdown strains are highlighted in blue and yellow, respectively, illustrating the range of survival phenotypes captured by the library. **(B)** Representative library members exhibiting at least a 10-fold difference in survival relative to the negative controls, Results for all qualifying members are shown in Supplementary Figure 1. Error bars represent the standard deviations from three biological replicates. **(C)** Survival curves of individually cultured knockdown strains used to validate the trends observed in the pooled RTK-seq screen. Error bars represent the standard deviations from three biological replicates. **(D)** Growth rates of selected knockdown strains normalized to that of the negative-control strain. Error bars represent the standard deviations from four biological replicates (*n* = 4). Statistical significance was assessed using an unpaired *t*-test relative to the negative control: *, *p* < 0.05; ns, *p* > 0.05. **(E)** qPCR quantification of TF repression in selected knockdown strains relative to the wild type strain.

To validate these results from this method, we first independently validated the survival phenotypes identified by RTK-seq. We performed conventional time-kill assays with selected TFKD strains. These individual assays recapitulated the corresponding survival phenotypes observed in the pooled RTK-seq screen, providing an orthogonal validation of the RTK-seq-derived trajectories (Figure 2C). To confirm that the identified TFDK strains have repressed TF expression, we further performed RT–qPCR on selected strains, including those with large changes in persistence level and randomly chosen library members. All tested TFKD strains showed 2-7-fold reduction in their corresponding TF expression (Figure 2E), confirming effective CRISPRi repression. Next, we aimed to rule out the effect of growth, which is known to influence persistence level during antibiotic treatment (Eng et al., 1991). We measured the exponential-phase growth rates of 10 TFKD strains with substantially higher or lower survival levels. Except for *hipB*, none of the selected TFKD strains exhibited significant changes in growth rate compared to the wild-type *E. coli* (Figure 2D), indicating that the observed survival phenotypes are not simply due to altered growth. The decrease in growth rate in the *hipB-*repressed strain was expected due to the accumulation of *HipA* toxin, which targets the GltX tRNA and causes translation to stall, resulting in ppGpp accumulation and slower cell growth (Kaspy et al., 2013; Page and Peti, 2016). Additionally, to confirm that the altered persistence phenotypes were indeed caused by repression of these TFs, we generated individual knockout strains of several representative TFs, hereafter referred to as TFKO strains. Each TFKO strain was subjected to an individual time-kill assay. The resulting time-kill curves closely matched those obtained from the multiplex screen (Supplementary Figure 2). Together, these results support that the identified TFs modulate pathways influencing ampicillin sensitivity and that our pooled assay reliably captures these effects.

To more quantitatively characterize the distinct killing dynamics captured by RTK-seq, we fitted the ampicillin time-kill trajectories to a biphasic exponential decay model (Carvalho et al., 2017):


$$y(t)=(1-A)e^{\left\{-K_{1}t\right\}}+Ae^{\left\{-K_{2}t\right\}}$$

Where *y*(*t*) represents the survival fraction at time (*t*), (*A*) represents the estimated persister cell fraction, and K_1_ and K_2_ designate the first-order rate constants for the initial fast-killing of sensitive cells and secondary slow-killing of persisters, respectively. Due to the limited data points in the slow killing phase, no statistically significant difference in K1 values were detected between the TFKD strain and the negative-control strains carrying non-targeting sgRNAs and any of the TFKD strains. This finding suggests that TFKD may not substantially alter the killing kinetics of sensitive cells, supporting that the observed differences among the time-kill curves reflect genuine persistence phenotypes arising from changes in the persister fraction or persister-killing kinetics, or both.

Our statistical analysis identified a total of 29 TFKD strains with significantly altered persister fraction (*araC, cra, lacI, hipB, lrhA, narP, mntR, metR, nadR, rcsA, fur, acrR, oxyR, galR, ycfQ, treR, mazF, nikR, tyrR, yoeB, ilvY, acrS, hypT, gadW, mazE, fhlA, mlc, aidB,* and *cytR*, Supplementary Table 9) and 5 TFKD strains with a significantly altered persister-killing kinetics (*acrR, ompR, relB, flhC,* and *torR*). Notably, *acrR* was the sole regulator present in both subsets, indicating its unique dual-modulatory role in governing both the abundance of persisters and their killing rate during ampicillin treatment.

### Known persistence mechanisms are recapitulated in the pooled TF knockdown library

Further examining the time-kill curves from RTK-seq captured multiple TFs previously implicated in antibiotic tolerance, supporting the idea that persistence can emerge from changes in multiple pathways including drug entry, stress adaptation, and toxin–antitoxin balance (Figure 3A). First, TFKD of *hipB*, which encodes the antitoxin component of the HipA–HipB toxin–antitoxin system, showed the biggest increase in persistence level. Repression of *hipB* is expected to increase free HipA activity, which can promote persistence by inhibiting essential cellular processes (Kaspy et al., 2013). In contrast, repression of *relB*, the antitoxin in the RelE–RelB system, maintained a normal growth rate and remained susceptible to antibiotics. RelE has recently been shown to reduce cellular fitness under chemical stress, which compromise stress buffering rather than simply increasing toxin activity, thus leading to reduced survival level (Davies et al., 2009; Keren et al., 2004). Second, regulators of oxidative stress responses, such as *oxyR* and *mntR*, were also identified, consistent with their roles in managing reactive oxygen species (ROS), which were shown to enhance survival during antibiotic treatment (Zhang et al., 2024; Dharmaraja, 2017; Ye et al., 2025). In addition, we identified multiple TFs that regulate envelope permeability and efflux pumps, including AcrAB–TolC-associated regulators such as *acrR* and *sdiA*, as well as regulators of the outer membrane porin OmpF such as *ompR* and *infA*. Because AcrAB–TolC and OmpF influence intracellular accumulation of ampicillin (Masi and Pagès, 2013; Ceccarelli et al., 2004; Rybenkov et al., 2021), repression of these regulators may alter drug influx or efflux, and thereby reducing cell survival. For example, TFKD of either *ompR* or *ihfA* releases the repression of OmpF, leading to enhanced antibiotic influx and decreasing persistence. To further confirm this pathway, we constructed an *ompF* overexpression strain (*ompF*^+^) to mimic the effects of *ompR* or *ihfA* knockdown and performed time-kill persistence assays. The *oxyR*^+^ strain also demonstrated decreased persistence level, similar to that observed in *ompR* and *ihfA* knockdown strains (Figure 3B). Thus, these results support a mechanistic link between these TFs and ampicillin tolerance. Overall, these observations demonstrate that one single experiment from RTK-seq can recapitulate multiple gene targets previously identified from numerous individual-strain studies.

**Figure 3:**
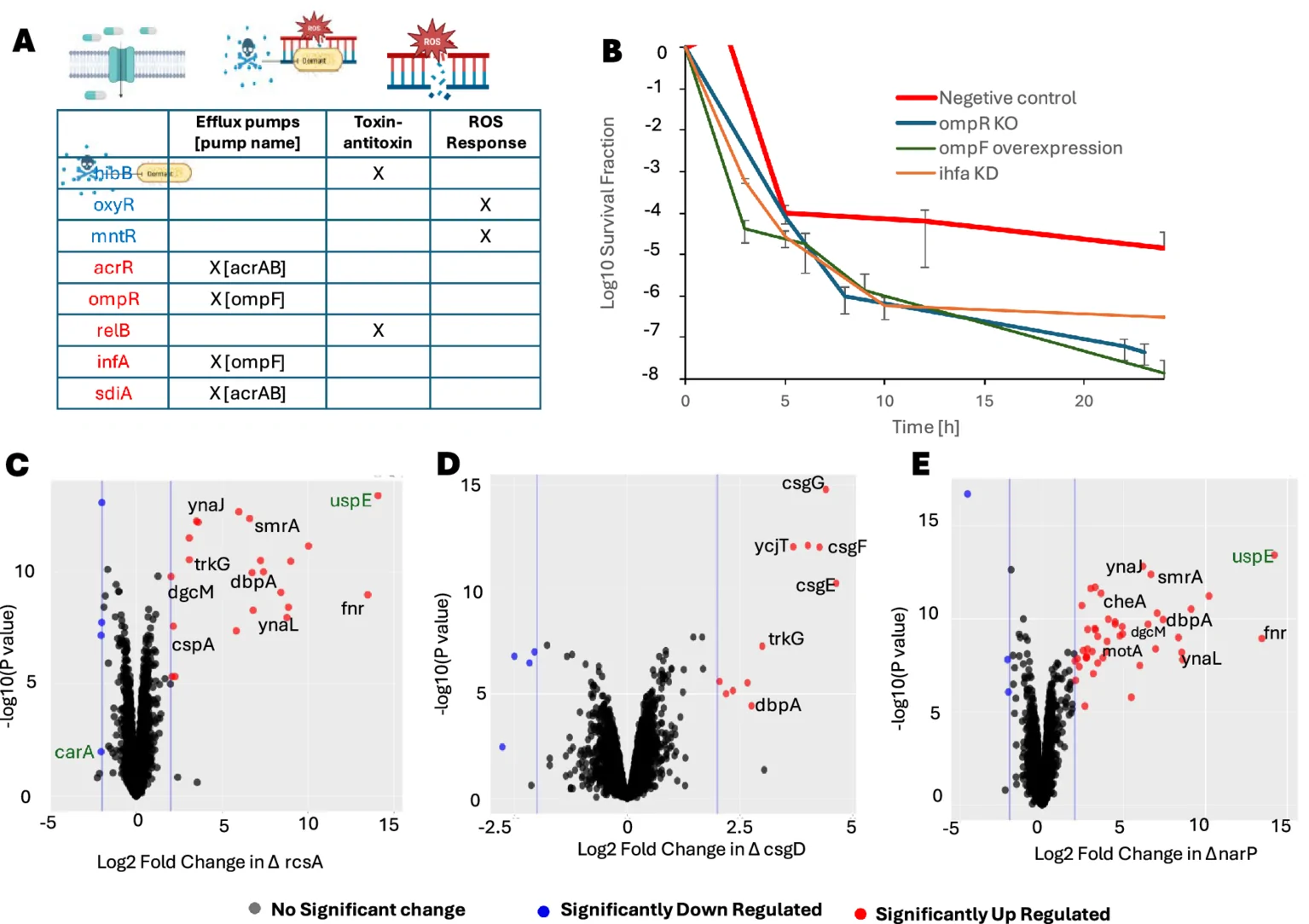
Mechanistic investigation of persistence phenotypes identified by RTK-seq. **(A)** Previously established persistence mechanisms recaptured by the RTK-seq screen. **(B)** Mechanistic analysis demonstrating that regulation of the *ompF* porin by OmpR and IhfA underlies the persistence phenotypes observed in the corresponding knockdown strains. **(C–E)** Transcriptomic volcano plots for the Δ*rcsA*, Δ*csgD*, and Δ*narP* strains respectively, showing genes with at least a twofold change in expression relative to the wild type. Labeled genes were selected for individual testing of their effects on persistence. **(D)**.

### Novel transcription factors and downstream effectors involved in persistence

Beyond regulators previously linked to persistence, we also identified TFs not previously associated with persistence, including *rcsA*, *narP*, and *csgD*. To determine which downstream genes may mediate the persistence phenotypes associated with these TFs, we performed transcriptomic profiling of these deletion mutants. Here, we used TF deletion strains instead of TFKD strains to minimize expression heterogeneity introduced by partial knockdown and to facilitate downstream analysis. Using a twofold change threshold relative to wild type, we obtained lists of candidate downstream genes whose altered expression may underlie the observed survival differences. Volcano plots revealed that most affected genes were upregulated in these mutants (Figures 3C–E), and that some genes were shared across multiple TF deletions, suggesting potential common mechanisms (Supplementary Figure 3, Supplementary Table 3).

Because none of the initially identified genes had previously been implicated in established persistence pathways, we decided to systematically test their contributions. For several genes with increased expression in one of the TF deletions, we constructed overexpression strains to recapitulate the TFKD-associated expression state. We prioritized operons for which multiple components were differentially expressed, as their combined activity may be required for the phenotype. For genes with decreased expression, we generated knockout strains to mimic their corresponding transcriptional effects. These candidates were then assayed for changes in persistence under ampicillin treatment. Among the tested genes, *csgE, uspE,* and *carA* were the only ones whose perturbation produced a change in persistence phenotype, with only *carA* being statistically significant (Supplementary Figure 4). The lack of a detectable persistence phenotype following perturbation of individual downstream candidates does not establish functional redundancy. Instead, these results suggest that persistence-associated transcriptional responses may involve multiple downstream factors or may depend on the magnitude and timing of their regulation. Differences between TF deletion and individual downstream-gene perturbation may also prevent complete recapitulation of the relevant physiological state.

### Differential behavior of the TF knockdown library across antibiotic classes

To extend the multiplex approach to other antibiotics and evaluate whether the effects of the identified TFKDs on *E. coli* persistence were antibiotic-specific, we applied the same workflow to two additional antibiotics with distinct modes of action: streptomycin, an aminoglycoside that targets the ribosome and inhibits protein synthesis, and ofloxacin, a fluoroquinolone that inhibits DNA replication. The library was again grown in M9 glucose medium and subjected to antibiotic treatment with time-resolved sampling and pooled sequencing (Figure 1).

Under streptomycin treatment, the library displayed broad sensitivity, with 60 TFKDs exhibiting decreased persistence relative to the wild type *E. coli* (Figure 4A, Supplementary Table 4). By contrast, ofloxacin treatment revealed only 10 TFKDs with pronounced changes, whereas the remaining strains showed survival trajectories similar to wild type *E. coli* (Figure 4B, Supplementary Table 5).

**Figure 4:**
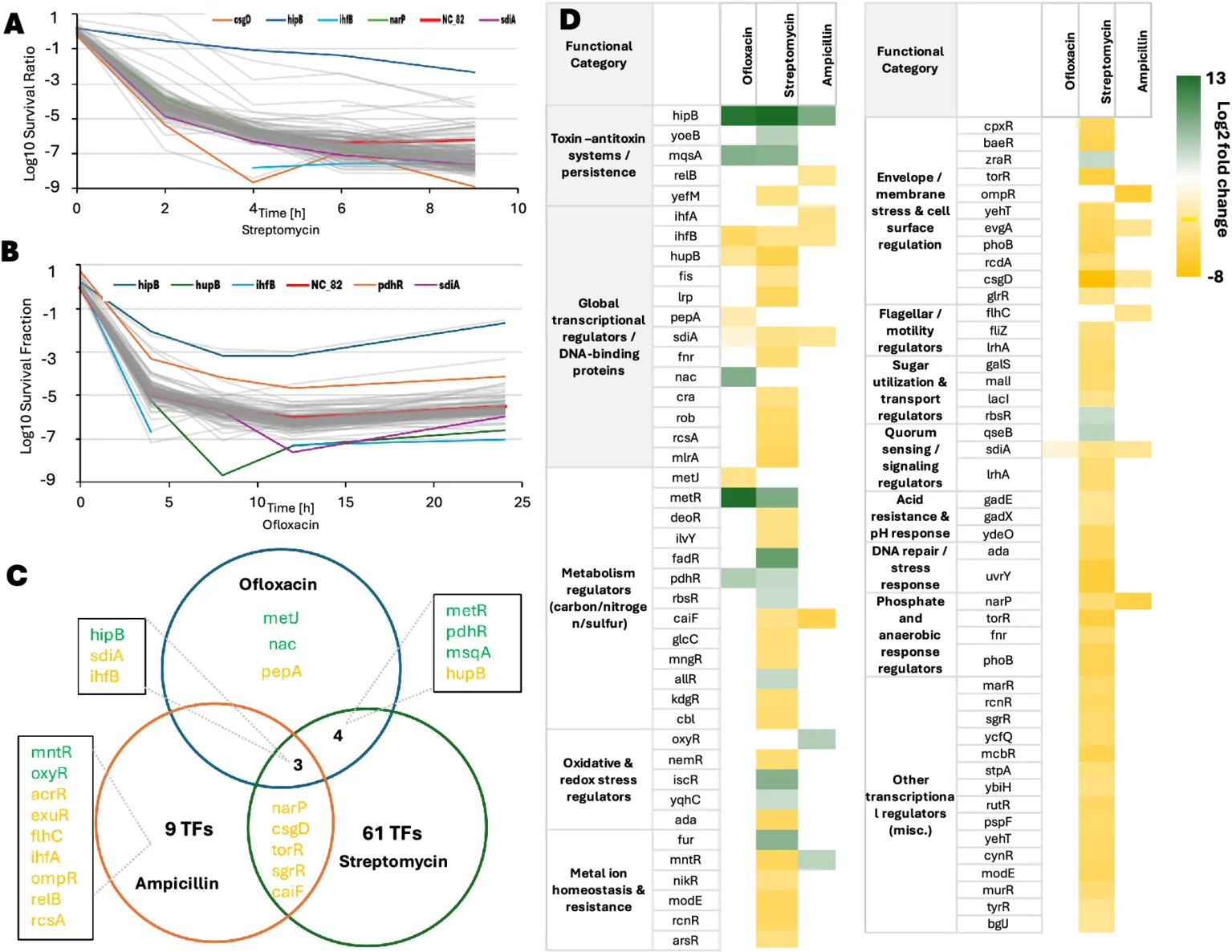
Comparison of RTK-seq persistence profiles across antibiotics. **(A)** Survival curves of RTK-seq library members following streptomycin treatment. **(B)** Survival curves of RTK-seq library members following ofloxacin treatment. **(C)** Diagram showing TFs whose perturbation produced at least a 10-fold change in persistence under more than one antibiotic treatment. **(D)** Heat map showing the log₂ fold change in persistence for TF perturbations that produced at least a 10-fold difference relative to the negative control. Genes are grouped according to their annotated biological or metabolic functions.

Using a threshold of greater than 10-fold difference in survival relative to wild type, we identified sets of TFs that substantially altered persistence under each antibiotic (Figure 4C, Supplementary Figures 5, 6). These TFs encompassed diverse functional categories, including toxin–antitoxin modules, oxidative stress responses, sugar utilization pathways, and DNA repair factors, illustrating the broad mechanistic coverage achievable with our approach. Comparisons across antibiotics revealed limited overlap: relatively few TFs affected persistence in more than one condition (Figure 4D, Supplementary Table 6). Across all three tested antibiotics, *hipB* was consistently identified as conferring increased persistence; meanwhile, *sdiA* and *ihfB* conferred decreased persistence, indicating that these TFs play essential roles in persister-associated regulatory networks. Streptomycin shared more TFs in common with ampicillin than with ofloxacin, likely because both antibiotics require active energy-consuming processes to cause cell death. Ofloxacin produced the smallest set of hits, with the fewest unique TFs. Among the four regulators that caused a decrease in persistence, 3 are duel-directional regulators (Nguyen Le Minh et al., 2016; Freundlich et al., 1992; Oberto et al., 2009; Houdt et al., 2006).

Collectively, these results indicate that the regulatory determinants of persistence are strongly condition-dependent and that our pooled TFKD library faithfully reports antibiotic-specific survival mechanisms rather than non-specific library artifacts. The RTK-seq method thus provides a versatile platform for mapping how global regulatory networks mediate tolerance to different antibiotic stresses.

## Discussion

This study establishes a generalizable, high-throughput functional genomics framework for dissecting the regulatory architecture of antibiotic persistence by systematically perturbing TFs and quantifying their continuous effects on killing dynamics at scale. By repurposing a pooled CRISPRi library in *E. coli*, we converted the classical, labor-intensive CFU-based persistence assay into a multiplexed, sequencing-based readout that yields high-resolution survival curves for hundreds of regulators in parallel. This RTK-seq approach bridges a long-standing methodological gap in the field, enabling the quantitative, high-throughput analysis of how every TFs influence bacterial persistence, which was previously constrained by low-throughput plating techniques.

A key strength of the RTK-seq framework is its capacity to resolve the continuous, multi-phasic kinetic trajectories that define true persistence. The close agreement between the RTK-seq reconstructed survival curves and conventional single-strain CFU validations confirms that barcode tracking faithfully captures population-level killing dynamics. Furthermore, RT-qPCR verified robust dCas9-mediated repression across tested strains, and growth profiling successfully ruled out confounding effects of general growth defects for the identified hits. The prominent exception was the *hipB* knockdown, which exhibited an expected growth rate reduction due to the toxic accumulation of uncoupled HipA, a well-characterized inducer of ppGpp-mediated translation arrest (Kaspy et al., 2013; Gerdes and Maisonneuve, 2012). Together, these internal validation controls affirm the specificity and robustness of the platform.

Our screen successfully recaptured multiple TFs with established roles in stress adaptation, validating the biological accuracy of the platform. The identification of *hipB*, *relB*, *oxyR*, and *mntR* emphasizes the interconnected roles of toxin-antitoxin activation and reactive oxygen species defense in promoting survival under bactericidal pressure (Zhang et al., 2024; Kaspy et al., 2013; Dharmaraja, 2017; Ye et al., 2025). Crucially, RTK-seq provided sufficient biological resolution to map drug transport dynamics. Knockdowns of *ompR* and *ihfA* led to a severe drop in ampicillin persistence, a phenotype we traced downstream to the derepression of the outer membrane porin OmpF and subsequent elevated antibiotic influx. This mechanistic link was validated by an *ompF* overexpression strain, which closely mirrored the accelerated killing kinetics of the *ompR* and *ihfA* knockdowns, illustrating how upstream TF screens can rapidly uncover specific structural determinants of drug accumulation.

Beyond recapitulating known survival pathways, RTK-seq uncovered novel regulatory nodes governing persistence, specifically flagging *rcsA*, *narP*, and *csgD*. Transcriptomic deconvolution of their downstream regulons led to the identification of *carA* as novel effectors whose disruption dramatically compromises ampicillin persistence. While single-gene validation of other highly differentially expressed targets like *csgE* and *uspE* did not yield statistically significant phenotypes on their own, this lack of single-gene causality likely highlights the complex, distributed architecture of bacterial persistence programs. Rather than relying on isolated “survival genes,” bacteria orchestrate multi-layered, redundant transcriptional responses that protect metabolic and structural homeostasis.

A central insight generated by this work is the strict condition-dependence of the persistence regulon. By applying RTK-seq across three distinct antibiotic classes, we observed remarkably little overlap among the TFs controlling survival under ampicillin, streptomycin, and ofloxacin. Streptomycin treatment triggered a wide spectrum of TF vulnerabilities, while ofloxacin yielded a minimal, specialized set of hits heavily biased toward dual-directional global regulators (Nguyen Le Minh et al., 2016; Freundlich et al., 1992; Oberto et al., 2009; Houdt et al., 2006). This divergence indicates that persistence is not a singular, universal physiological state, but a collection of distinct metabolic and structural adaptations tailored to counter specific drug-induced cellular damage. It is also worth noting that all bacterial cultures in this study were grown in defined M9 glucose medium to provide a reproducible physiological environment. Clinically relevant bacteria growing under different nutrient conditions may exhibit distinct growth rates, metabolic states, and regulatory activities, which could lead to different persistence phenotypes and outcomes. These condition-specific regulatory maps have immediate clinical and industrial utility. From a therapeutic standpoint, mapping the precise TFs required to survive specific bactericidal agents provides a blueprint for rational adjuvant design. For example, chemical inhibitors targeting OmpR or specific stress-response regulators could be co-administered with beta-lactams to artificially stimulate drug influx or prevent the activation of protective dormant states, effectively sensitizing recalcitrant persisters subpopulations in chronic or recurrent infections.

From an industrial biotechnology perspective, the RTK-seq platform can serve as a high-throughput diagnostic engine for strain optimization. The cellular stresses encountered by production hosts in large-scale bioreactors, such as local organic acid accumulation, oxidative bursts, and toxic biofuel levels, highly parallel the physiological disruptions imposed by antibiotics (Jiang et al., 2017; Miller et al., 2009; Bai et al., 2022; Jiang et al., 2018). By substituting antibiotics with industrial stressors (e.g., high titers of isobutanol or furfural), RTK-seq can rapidly identify TFs whose knockdown or overexpression enhances survival and metabolic homeostasis. This predictive screening capacity can directly guide the engineering of more resilient microbial chassis, minimizing biomass loss, and maximizing productivity during extended fermentation runs.

Meanwhile, our current study has several limitations that suggest paths for future development. First, Because CRISPRi produces variable and often incomplete repression, residual transcription factor expression may remain sufficient to maintain downstream regulatory states, potentially masking phenotypes that depend on crossing a critical level of TF activity (Wang et al., 2018; Wall et al., 2004; Rousset et al., 2018). Utilizing variable-strength promoters or inducible systems could help chart these nonlinear effects (Liu et al., 2013; Davis et al., 2011). Second, this study was restricted to exponential-phase cultures in defined media; extending RTK-seq to stationary-phase populations, nutrient-depleted regimes, or host-mimicking environments will be essential for capturing clinically realistic persistence landscapes. Additionally, because the RTK-seq library contains a single sgRNA per transcription factor, guide-specific differences in CRISPRi activity, off-target effects, and polar effects cannot be completely excluded for every regulator. However, previous genome-scale CRISPRi screens in *E. coli* showed the off-target (10.7%) and polarity effect (8.0%) probabilities are generally low. Our sgRNA design further eliminated bad-seed effects that cause off-target in the previous CRISPRi screens; thus, the expected false positive rate should be substantially low. Even if off-target or polar effects unexpectedly perturbed a gene, its possibility to cause persistence change is expected to be lower than that from a TF. Finally, our downstream follow-ups focused primarily on single-gene disruptions. Integrating RTK-seq datasets with multi-omic networks and constraint-based metabolic models will be necessary to fully map the flow from regulatory inputs to survival outputs.

In conclusion, the RTK-seq platform provides a scalable, versatile, and highly quantitative framework that links TFs to time-resolved killing kinetics. By exposing the drug-specific topology of bacterial tolerance, this method offers a powerful tool for deciphering microbial survival mechanisms, with clear applications for both the engineering of robust industrial strains and the development of next-generation antimicrobial therapies.

## Methods

### Bacterial strains, plasmids, and culture conditions

The RTK-seq library was derived from the CRISPRi transcription-factor library previously developed for PPTP-seq, which contains a single sgRNA targeting each of the 183 transcription factor genes in *E. coli* (Han et al., 2023) and transformed into the *E. coli* strain FR-E01, which carries a chromosomally integrated dCas9 (Rousset et al., 2018). All resulting transformant colonies (approximately 5 × 10^6^ individual clones as estimated by serial dilution on small LB agar plates) were pooled and transferred directly into Luria-Bertani (LB) medium supplemented with kanamycin (Kan). The transformed cell mixtures were grown for 2–3 h. The strain library was then used to prepare glycerol stocks by flash-frozen in liquid nitrogen and stored at −80 °C until further use. Full list of strains used can be found it Supplementary Table 8.

### Controlled logarithmic growth and acclimation

For each antibiotic condition, RTK-seq was performed using three biological replicates with unique barcodes for tracking. For each antibiotic, all barcoded DNA were pooled together for sequencing. For each replicate, 100 μL of strain library glycerol stock was used to inoculate 5 mL of LB medium supplemented with Kan and grown for 3 h at 37 °C. Next, 400 μL of this culture was used to inoculate 5 mL of M9-glucose medium containing 1 μM anhydrotetracycline (aTc) and grown for an additional 3 h. The culture was then diluted into 50 mL of fresh M9-glucose medium supplemented with aTc and Kan in a 250 mL Erlenmeyer flask to an initial OD_600_ of 4 × 10^−4^ and grown until it reached an OD_600_ of 0.4.

At an OD_600_ of 0.4, duplicate 1 mL culture samples were harvested, and the remaining cultures were challenged with antibiotics: ampicillin (100 μg/mL), streptomycin (35 mg/L), or ofloxacin (5 mg/L). One 1 mL aliquot was pelleted and stored at −80 °C for subsequent miniprep. From the second aliquot, 200 μL was serially diluted in phosphate-buffered saline (PBS) in a 96-well plate, and 5 μL aliquots of each dilution were plated on LB-Kan agar. After overnight incubation at 37 °C, colonies were counted to determine colony-forming units (CFU) per ml of culture. The remaining 800 μL of the harvested culture was plated onto a 500 cm^2^ LB-Kan agar plate and incubated overnight at 37 °C.

At designated time points after antibiotic addition, culture aliquots were harvested. The sampled volume was adjusted as needed to ensure that at least 20,000 colonies were obtained on each large plate. At each time point, an aliquot of the liquid culture was collected and centrifuged at 6,500 × g for 5 min at 4 °C and washed twice with PBS to remove residual antibiotic. The washed pellet was resuspended in 500 μL PBS. A 50 μL portion was serially diluted in PBS in a 96-well plate, and 5 μL aliquots of each dilution were plated on LB-Kan agar for CFU counting. The remaining resuspended pellet was plated onto a 500 cm^2^ LB-Kan agar plate and incubated overnight at 37 °C. All colonies on the large plates were subsequently harvested by resuspension in 20 mL PBS.

### Library preparation and next-generation sequencing

Library preparation and sequencing were performed following previous methods with modifications (Han et al., 2023; Schmitz and Zhang, 2021). Specifically, total plasmid DNA was extracted from both the pre-antibiotic liquid-culture cell pellets or the pooled solid-media colony suspensions using a commercial plasmid miniprep kit according to the manufacturer’s instructions (iNtRON Biotechnology DNA-spin). For library amplification, 50 ng of purified plasmid DNA from each discrete time point was used as the template in a 20-cycle PCR using the KAPA HiFi PCR Kit (Kapa Biosystems) with primers prYH071 and prYH087 (Supplementary Table 7). Amplicons were purified using a Zimo DNA Clean and Concentrator kit and subsequently PCR amplified with Illumina i5 and i7 barcode primers for a secondary low-cycle PCR. This 8-cycle PCR was executed using the KAPA HiFi enzyme with indexed primers (Supplementary Table 7).

Each sample was pooled to have an equal percentage of the final pool and a molar concentration of 40 nM. The final balanced library pool was subjected to next-generation sequencing using partial lanes of a NovaSeq S4 XP platform with 2 × 150 bp paired-end chemistry at the Genome Technology Access Center within the McDonnell Genome Institute. Sequencing was performed to an average depth of 7.7 M reads per sample timepoint, corresponding to approximately 42,000 reads per library member, assuming an even distribution across all library members (see Supplementary Table 1 for actual reads).

### Curve calculations

Barcodes were used to assign sequencing reads to individual library members and determine their read counts at each time point. For each library member, the read fraction, α_it_, was calculated by dividing the number of reads of TF-*i* at time t by the total number of reads, *R*, at time *t*.


$$\alpha_{\mathrm{it}}=(\frac{r_{\mathrm{it}}}{R_{t}})$$ (1)

Read fractions at every time point were then normalized to the read fraction at time 0 before antibiotic treatment to get the pretreatment fraction β_it_.


$$\beta_{\mathrm{it}}=\frac{\alpha_{\mathrm{it}}}{\alpha_{i,t=0}}$$ (2)

The shift in abundance between liquid culture and colony size variance on agar was then calculated as ω_i_. For each TF, this was calculated by dividing the read fraction before antibiotic treatment from the liquid media by the read fraction before antibiotic treatment from colonies grown on the agar plate.


$$\omega_{i}=\frac{\alpha_{i,t=0\;\text{liquid culture}}}{\alpha_{i,t=0\;\text{agar plate}}}$$ (3)

The final survival fraction, ε _it_, was then calculated for each TF at each timepoint by dividing the normalized read fraction by the colony size variance and multiplying by survival fraction δ_t_ of the library at each timepoint from the serial dilution survival curve.


$$\varepsilon_{\mathrm{it}}=\frac{\beta_{\mathrm{it}}}{\omega_{i}}\;\delta_{t}$$ (4)

### Transcriptomics

TFKD strains and the wild-type control were grown in biological triplicate in M9 minimal medium supplemented with glucose at 37 °C with shaking at 250 rpm. Cultures were inoculated from 4-h M9 glucose precultures and grown to the exponential phase (7 generations). Cells were harvested when cultures reached an OD_600_ of approximately 0.4.

For RNA extraction, culture volumes corresponding to approximately 1 × 10 (Windels et al., 2019) cells were collected by centrifugation and immediately processed. Total RNA was isolated using the Qiagen RNeasy Bacterial RNA Kit according to the manufacturer’s instructions. Genomic DNA contamination was removed by on-column DNase digestion using the RNase-Free DNase Set (Qiagen). RNA concentration and purity were assessed using a Nanodrop, and RNA integrity was evaluated prior to library preparation.

Purified RNA samples were submitted to the Genome Technology Access Center within the McDonnell Genome Institute for library preparation, next-generation sequencing, and primary bioinformatic processing. Ribosomal RNA was depleted prior to library construction, and sequencing libraries were generated according to the provider’s standard bacterial RNA-seq workflow. Libraries were sequenced on an Illumina platform to generate paired-end reads.

Raw sequencing reads were subjected to quality control, adapter trimming, and filtering by the sequencing provider. High-quality reads were aligned to the *E. coli* reference genome, and gene-level read counts were generated. Differential gene expression analysis was performed by comparing each TFKO strains to the wild-type control. Gene expression changes were calculated as log2 fold changes, and statistical significance was determined using the provider’s standard RNA-seq analysis pipeline. Genes exhibiting a minimum two-fold change in expression relative to wild type were considered differentially expressed and were selected for downstream analysis. Differentially expressed genes were further analyzed to identify candidate pathways and downstream targets potentially associated with altered persistence phenotypes observed in antibiotic survival assays.

### Growth rate

Cells at OD_600_ 0.4 growing in LB medium were collected and diluted 10-fold into fresh medium in 96-well plates. The plates were then transferred to an Infinite F200PRO plate reader (TECAN, Männedorf, Switzerland), and OD_600_ values were automatically over time. Cell growth rates were calculated from the slope of the linear region corresponding to exponential growth. The resulting growth rates were normalized to that of the wild-type strain.

#### qPCR

To quantify gene expression, three independent bacterial colonies were inoculated into LB medium and cultured overnight. The resulting cultures were diluted 200-fold into 5 mL of M9 glucose medium and grown for 1 h. Transcription was then induced by adding anhydrotetracycline (aTc) to a final concentration of 1 μM, followed by an additional 2 h of incubation. To maintain exponential growth, the cultures were subsequently diluted 900-fold into fresh M9 glucose medium containing 1 μM aTc and harvested upon reaching an OD₆₀₀ of 0.1. Total RNA was isolated from 2 mL of each culture using the Quick-RNA kit (Zymo Research) according to the manufacturer’s protocol. Residual genomic DNA was removed by in-column DNase treatment (Zymo Research), and cDNA was synthesized using the RevertAid First Strand cDNA Synthesis Kit (Thermo Fisher Scientific). Quantitative PCR (qPCR) was conducted on a QuantStudio 3 Real-Time PCR System (Applied Biosystems) using PowerTrack SYBR Green Master Mix (Thermo Fisher Scientific). Target-gene expression was normalized to the constitutively expressed reference gene *dnaK*, and relative fold changes were determined using the comparative 2^-∆∆CT^ method (Schmittgen and Livak, 2008).

## Data Availability

The RNA-sequencing data generated in this study has been deposited in the Gene Expression Omnibus database under accession code GSE347678 [https://www.ncbi.nlm.nih.gov/geo/query/acc.cgi?acc=GSE347678]. Other datasets presented in this study can be found in the article or Supplementary material.
