# Supplementary material for: Regulatory time-kill sequencing maps antibiotic-specific transcriptional control of Escherichia coli persistence at genome scale
Source: Front Microbiol. 2026 Sep 24;17:1950209. doi: 10.3389/fmicb.2026.1950209 (PMC13646166; doi:10.3389/fmicb.2026.1950209)
Supplement: Supplementary file 1 [file Data_Sheet_1.docx]

**Regulatory Time-Kill Sequencing Maps Antibiotic-Specific Transcriptional Control of *Escherichia coli* Persistence at Genome Scale**

**Authors:** Alden Filko^1^, Yichao Han,^1^ Fuzhong Zhang^1-3^ *

Affiliations:

^1^Department of Energy, Environmental and Chemical Engineering,

^2^Division of Biological & Biomedical Sciences,

^3^Institute of Materials Science & Engineering,

Washington University in St. Louis, Saint Louis, Missouri, USA.

*e-mail: [fzhang@seas.wustl.edu](mailto:fzhang@seas.wustl.edu)

Survival fraction

| Time [h] | sdiA | relB | ompR | oxyR | narP | mntR | ihfA | ihfB | hipB | flhC | evgA | csgD | caiF | acrR | NC_84 |
| --- | --- | --- | --- | --- | --- | --- | --- | --- | --- | --- | --- | --- | --- | --- | --- |
| 0 | 1 | 1 | 1 | 1 | 1 | 1 | 1 | 1 | 1 | 1 | 1 | 1 | 1 | 1 | 1 |
| 4 | 0.000108873 | 0.000686794 | 0.00010206 | 0.00130239 | 0.00270088 | 0.0025805 | 0.000180992 | 0 | 0.0146031 | 0.000146899 | 4.18834e-05 | 3.71222e-05 | 0.000111583 | 0.000202172 | 0.00017949 |
| 6 | 3.60508e-06 | 0.000300889 | 4.75689e-05 | 0.00219153 | 0.00275347 | 0.00246173 | 2.28528e-05 | 3.77098e-05 | 0.0350453 | 0.00014717 | 5.8413e-05 | 0 | 2.16033e-05 | 0.000161109 | 9.10776e-05 |
| 12 | 4.35861e-06 | 6.91757e-06 | 4.24e-06 | 0.000391199 | 0.00125064 | 0.000916511 | 6.67764e-06 | 8.6159e-07 | 0.0325585 | 3.62631e-05 | 8.98332e-05 | 7.76165e-06 | 2.15161e-05 | 2.76342e-05 | 6.15374e-05 |
| 24 | 2.51461e-06 | 2.51583e-06 | 3.26867e-07 | 0.000651739 | 0.000347211 | 0.000413289 | 1.87364e-06 | 2.2141e-06 | 0.00589795 | 2.05652e-06 | 2.30969e-06 | 2.45627e-06 | 5.77261e-07 | 2.07583e-07 | 1.89386e-05 |

**Supplementary Figure1**: All library members with a 10-fold change in survival compared to the negative control from RTK-seq performed in ampicillin. NC_84 represents the negative control with non-targeting sgRNA.

ihfA

Survival Fraction

| Time [h] | Knockout |
| --- | --- |
| 0 | 1 |
| 3 | 0.000242766 |
| 8 | 3.83242e-06 |
| 11 | 9.69597e-06 |
| 24 | 2.55431e-07 |

| Time [h] | Knockdown |
| --- | --- |
| 0 | 1 |
| 3 | 0.000605128 |
| 5 | 2.60633e-05 |
| 10 | 5.86727e-07 |
| 24 | 3.16742e-07 |

| Time [h] | Sequincing |
| --- | --- |
| 0 | 1 |
| 4 | 0.000180992 |
| 6 | 2.28528e-05 |
| 12 | 6.67764e-06 |
| 24 | 1.87364e-06 |

| Time [h] | NC |
| --- | --- |
| 0 | 1 |
| 2 | 2.58278 |
| 5 | 0.000102601 |
| 12 | 6.26777e-05 |
| 24 | 1.41905e-05 |

ompR

Survival Fraction

| Time[h] | Knockout |
| --- | --- |
| 0 | 1 |
| 5 | 7.98942e-05 |
| 8 | 9.87302e-07 |
| 22 | 5.9612e-08 |
| 23 | 4.50211e-08 |

| Time[h] | Knockdown |
| --- | --- |
| 0 | 1 |
| 4 | 0.000280933 |
| 17 | 1.73759e-06 |
| 24 | 5.27482e-07 |

| Time[h] | Sequincing |
| --- | --- |
| 0 | 1 |
| 4 | 0.00010206 |
| 6 | 4.75689e-05 |
| 12 | 4.24e-06 |
| 24 | 3.26867e-07 |

| Time[h] | NC |
| --- | --- |
| 0 | 1 |
| 2 | 2.58278 |
| 5 | 0.000102601 |
| 12 | 6.26777e-05 |
| 24 | 1.41905e-05 |

Negetive control

Survival Fraction

| Time [h] | Sequencing |
| --- | --- |
| 0 | 1 |
| 4 | 0.00017949 |
| 6 | 9.10776e-05 |
| 12 | 6.15374e-05 |
| 24 | 1.89386e-05 |
| 48 | 0.000194623 |
| 60 | 3.58698e-05 |

| Time [h] | knockdown |
| --- | --- |
| 0 | 1 |
| 2 | 2.58278 |
| 5 | 0.000102601 |
| 12 | 6.26777e-05 |
| 24 | 1.41905e-05 |
| 50 | 0.000275 |
| 60 | 2.60586e-06 |

csgD

Survival Fraction

| Time [h] | Knockdown |
| --- | --- |
| 0 | 1 |
| 4 | 2.02862e-05 |
| 6 | 1.25331e-05 |
| 12 | 6.14077e-08 |
| 24 | 5.11671e-08 |

| Time [h] | Sequincing |
| --- | --- |
| 0 | 1 |
| 4 | 3.71222e-05 |
| 12 | 7.76165e-06 |
| 24 | 2.45627e-06 |

| Time [h] | NC |
| --- | --- |
| 0 | 1 |
| 2 | 2.58278 |
| 5 | 0.000102601 |
| 12 | 6.26777e-05 |
| 24 | 1.41905e-05 |

| Time [h] | Knockout |
| --- | --- |
| 0 | 1 |
| 3 | 0.000369231 |
| 8 | 1.15385e-05 |
| 11 | 3.07692e-07 |
| 24 | 5.63962e-08 |

**Supplemental Figure 2**: Time-kill curves deconvoluted from RTK-seq (labeled as “Sequencing”) compared to those obtained from individual measurements using TFKD (labeled as “Knockdown”) and TFKO (labeled as “Knockout”) strains in ampicillin for representative TFs.

**Supplementary Figure 3:** Transcriptomic heat map showing overlapping differentially expressed genes among the knockout strains.

14.2

-4.6

Log2 fold change

*

NS

Log10 Survival Fraction

|  | wild type ∆ carA csgEFG uspE wild type ∆ carA csgEFG uspE fnr trkG ynaJ ∆ arrs FR-E01 ∆ narP csgE grcA uspE fre01 csgE motA B, cheA,W flhDC dgcM smrA dbpA ,ynal grcA cspA uspE zntB ∆ mrcB ∆ abgT ∆ ttc |
| --- | --- |
| wild type | -4.54084 |
| ∆ carA | -6.56849 |
| csgEFG | -5.38564 |
| uspE | -5.98973 |
| fnr | -4.35828 |
| trkG | -4.57184 |
| ynaJ | -4.76614 |
| ∆ arrs | -4.88768 |
| FR-E01 | -4.54084 |
| grcA | -4.3906 |
| csgE | -3.7733 |
| motA B, cheA,W | -3.58711 |
| flhDC | -3.51848 |
| dgcM | -3.92196 |
| smrA | -3.5988 |
| dbpA ,ynal | -3.75325 |
| cspA | -3.76133 |
| zntB | -4.71376 |
| ∆ mrcB | -4.95411 |
| ∆ abgT | -5.17599 |
| ∆ ttcA | -4.51136 |

**Supplementary Figure 4:** Survival fraction at 24h of ampicillin treatment for strains with gene overexpression or gene knockout. Individual values from three biological replicates (*n* = 3) and the corresponding means ± s.d. are shown on a log₁₀ scale. Statistical significance was assessed using a two-tailed Welch’s *t*-test comparing each mutant independently with wild-type control, *P =0.0096, *, ns P>0.05*

Survival Fraction

| Time [h] | ada | allR | arsR | baeR | bglJ | caiF | cbl | cpxR | cra | csgD | cynR | dcuR | deoR | evgA | fadR | fis | fliZ | fnr | fur | gadE |
| --- | --- | --- | --- | --- | --- | --- | --- | --- | --- | --- | --- | --- | --- | --- | --- | --- | --- | --- | --- | --- |
| 0 | 0.941447 | 0.999591 | 0.941462 | 1.06464 | 0.938733 | 1.07364 | 1.06396 | 0.929315 | 1.02051 | 0.591471 | 0.913576 | 1.00837 | 0.926951 | 0.991253 | 1.04239 | 1.29766 | 1.03787 | 0.972152 | 27.5299 | 0.99672 |
| 2 | 6.78031e-05 | 3.58241e-05 | 9.82992e-05 | 7.05613e-06 | 5.16091e-05 | 0 | 3.29736e-05 | 3.23275e-05 | 9.54561e-05 | 4.24731e-06 | 1.92463e-05 | 6.7082e-05 | 3.67546e-05 | 2.07838e-05 | 0.000152322 | 0.000108434 | 0.000177563 | 5.42718e-05 | 7.5069 | 5.85155e-05 |
| 4 | 1.42974e-06 | 1.23745e-06 | 1.33653e-06 | 8.16165e-07 | 1.4717e-06 | 4.20966e-07 | 1.26281e-06 | 1.00396e-06 | 1.97795e-06 | 2.15127e-09 | 5.4758e-07 | 1.90912e-06 | 1.67411e-06 | 5.91221e-07 | 6.64187e-06 | 3.87732e-06 | 1.89933e-06 | 3.48927e-07 | 0.00169819 | 1.60675e-06 |
| 6 | 1.68287e-08 | 4.56011e-07 | 3.40337e-07 | 6.17297e-08 | 2.02463e-07 | 3.60287e-08 | 2.08309e-07 | 4.47289e-08 | 4.81229e-07 | 1.31199e-07 | 1.27931e-07 | 1.2504e-06 | 1.30068e-07 | 1.32166e-08 | 2.17931e-05 | 8.80092e-07 | 4.67478e-07 | 2.34464e-07 | 0.00389272 | 2.83379e-07 |
| 9 | 1.11307e-08 | 3.86378e-06 | 2.08543e-08 | 7.7314e-09 | 3.25927e-08 | 2.47091e-08 | 1.48453e-08 | 9.96558e-09 | 2.36332e-08 | 1.29052e-09 | 1.2303e-08 | 1.21943e-05 | 2.36174e-08 | 9.68343e-09 | 0.000181746 | 2.99274e-08 | 1.96559e-08 | 1.48092e-08 | 4.55511e-05 | 3.23434e-08 |

**Supplemental Figure 5**: Time-kill curves of all TFs with more than 10-fold change from the negative control as obtained from RTK-seq performed in streptomycin.

Survival Freaction

| Time [h] | hipB | hupB | ihfB | metJ | metR | mqsA | nac | pdhR | pepA | sdiA | NC |
| --- | --- | --- | --- | --- | --- | --- | --- | --- | --- | --- | --- |
| 0 | 1.64554 | 0.916818 | 1.21805 | 2.15205 | 1.32856 | 269.28 | 2.46766 | 5.02833 | 1.70294 | 0.906952 | 0.930845 |
| 4 | 0.00916529 | 5.71314e-06 | 2.04516e-07 | 1e-12 | 0.00570024 | 0.000494788 | 0.00134406 | 0.000474472 | 4.5991e-06 | 1.00185e-05 | 1.39322e-05 |
| 8 | 0.000662159 | 2.04545e-09 | 1e-12 | 1e-12 | 0.00136492 | 1e-12 | 9.51006e-06 | 6.49186e-05 | 4.09391e-07 | 1.77229e-06 | 2.55053e-06 |
| 12 | 0.000646574 | 4.67403e-08 | 5.3604e-08 | 7.04943e-07 | 0.00109716 | 0.000146722 | 3.34577e-05 | 2.21699e-05 | 8.84968e-08 | 2.35511e-08 | 1.01652e-06 |
| 24 | 0.0211827 | 2.57156e-07 | 8.96381e-08 | 2.08212e-07 | 0.0289547 | 0.000449952 | 0.000484852 | 7.00423e-05 | 4.63761e-07 | 1.04933e-06 | 2.53753e-06 |

**Supplemental Figure** **6**: Time-kill curves of all TFs with more than 10-fold change from the negative control as obtained from RTK-seq performed in ofloxacin.
